# Fabrication and assessment of potent anticancer nanoconjugates from chitosan nanoparticles, curcumin, and eugenol

**DOI:** 10.3389/fbioe.2022.1030936

**Published:** 2022-12-08

**Authors:** Mohsen M. El-Sherbiny, Rawan S. Elekhtiar, Mohamed E. El-Hefnawy, Hoda Mahrous, Sultan Alhayyani, Soha T. Al-Goul, Mohamed I. Orif, Ahmed A. Tayel

**Affiliations:** ^1^ Department of Marine Biology, King Abdulaziz University, Jeddah, Saudi Arabia; ^2^ Department of Fish Processing and Biotechnology, Faculty of Aquatic and Fisheries Sciences, Kafrelsheikh University, Kafr el-Sheikh, Egypt; ^3^ Department of Chemistry, Rabigh College of Sciences and Arts, King Abdulaziz University, Jeddah, Saudi Arabia; ^4^ Genetic Engineering and Biotechnology Research Institute, University of Sadat City, Sadat, Egypt; ^5^ Department of Marine Chemistry, King Abdulaziz University, Jeddah, Saudi Arabia

**Keywords:** anticancer, nanochitosan, nanocomposites, cytotoxicity, *in vitro*

## Abstract

In cancer management and control, the most challenging difficulties are the complications resulting from customized therapies. The constitution of bioactive anticancer nanoconjugates from natural derivatives, e.g., chitosan (Ct), curcumin (Cur), and eugenol (Eug), was investigated for potential alternatives to cancer cells’ treatment. Ct was extracted from *Erugosquilla massavensis* (mantis shrimp); then, Ct nanoparticles (NCt) was fabricated and loaded with Cur and/or Eug using crosslinking emulsion/ionic-gelation protocol and evaluated as anticancer composites against CaCo2 “colorectal adenocarcinoma” and MCF7 “breast adenocarcinoma” cells. Ct had 42.6 kDa molecular weight and 90.7% deacetylation percentage. The conjugation of fabricated molecules/composites and their interactions were validated *via* infrared analysis. The generated nanoparticles (NCt, NCt/Cur, NCt/Eug, and NCt/Cur/Eug composites) had mean particle size diameters of 268.5, 314.9, 296.4, and 364.7 nm, respectively; the entire nanoparticles carried positive charges nearby ≥30 mV. The scanning imaging of synthesized nanoconjugates (NCt/Cur, NCt/Eug, and NCt/Cur/Eug) emphasized their homogenous distributions and spherical shapes. The cytotoxic assessments of composited nanoconjugates using the MTT assay, toward CaCo2 and MCF7 cells, revealed elevated anti-proliferative and dose-dependent activities of all nanocomposites against treated cells. The combined nanocomposites (NCt/Eug/Cur) emphasized the highest activity against CaCo2 cells (IC_50_ = 11.13 μg/ml), followed by Cur/Eug then NCt/Cur. The exposure of CaCo2 cells to the nanocomposites exhibited serious DNA damages and fragmentation in exposed cancerous cells using the comet assay; the NCt/Eug/Cur nanocomposite was the most forceful with 9.54 nm tail length and 77.94 tail moment. The anticancer effectuality of innovatively combined NCt/Cur/Eug nanocomposites is greatly recommended for such biosafe, natural, biocompatible, and powerful anticancer materials, especially for combating colorectal adenocarcinoma cells, with elevated applicability, efficiency, and biosafety.

## 1 Introduction

The use of nanotechnology in health care and medications has received much interest from investigators, particularly in cancer treatments; nanomaterials can effectively deliver bioactive molecules/drugs to targeted cells with minimal harm to healthy cells (Oh et al., 2021). Nanoparticle-based medications could augment the solubility, bioavailability, stability, and drug half-lives of various chemotherapeutic agents; they could also intensify drug accumulation inside the targeted cells/tissues (Ojeda-Hernández et al., 2020). The nanoparticles (NPs), by their minute sizes and capability to coat/carry other molecules, simplify hydrophobic drug delivery to targeted sites/cells with diminished rejection from the immune system (Jesus et al., 2020). Moreover, NPs could reinforce drug accumulation inside cancerous cells by intensifying cells’ retention/permeability, which augments therapy’s efficacy and diminishes target–ligand interactions (Zhao et al., 2018).

Cancers are ailments that could impair any body organ/tissue; they are the second globally leading cause of human demise with ≥16.5% of total deaths killing ≥10 million people annually in 2020 (World Health Organization, 2021). Colorectal and breast cancers are the most disastrous malignant causes of global mortality; they caused ∼1.6 million deaths and comprised 21.7% of the recently diagnosed cancer patients in 2020 (Sung et al., 2021). The colorectal adenocarcinoma (CaCo2) epithelial cells are presumably the most used *in vitro* models for investigating intestinal functions, whereas the breast cancer (MCF7) epithelial cells have the reference gene expression profiles for tracing the functions of anticancer candidates (van der Zande et al., 2016). One of the cancers’ common traits is metastasis; the speedy irregular cell formation that can multiply beyond the ordinary borders, allowing it to invade neighboring segments of body and migrate to further tissues (Macha et al., 2019). The usual cancer management includes surgeries, radiotherapies, and chemotherapies; these treatments can regularly cause numerous complications and side effects for patients (Demain and Vaishnav, 2011; Ahmad et al., 2021). Medicinal/aromatic plants and their constituents could provide effectual solutions for aforementioned contradictions in cancer management (Wang et al., 2012; Ahmad et al., 2021). The phytoconstituents were used in ancient traditional medications for numerous diseases and were the basis of many topical medicines (Kooti et al., 2017). Due to their elevated efficiency, biocompatibility, cost-effectiveness, and minimal side effects, phytocompounds were recurrently used as effective inhibitors, treatments, and remedies for cancer-associated diseases (Wang et al., 2012; Kooti et al., 2017; Ahmad et al., 2021). The polymeric NPs validated their performance for effectual drug carrying, delivery, and targeting; they could be easily synthesized and had biodegradable, non-immunogenic, biocompatible, non-toxic, water-soluble, and cost-effective attributes (Jesus et al., 2019). The polymeric nanoparticles (including chitosan and other seaweed polysaccharides) have a particle diameter range of 1–1,000 nm (Demain and Vaishnav, 2011; Zhao et al., 2018; Macha et al., 2019; Jesus et al., 2020), which differs from nanometals that frequently have a diameter range of 1–100 nm.

Chitosan (Ct), the deacetylated derivative of chitin, can be obtained from fungal mycelium, crustaceans’ shells, insect skeletons, or plant materials (Rinaudo, 2006). Ct can be smoothly transformed into nanoforms (mainly *via* the self-assembly, polyelectrolyte complexes, and ionic cross-linking methods), which makes chitosan nanoparticles (NCt) the ideal nanocarriers for various drugs, with prominent biodegradability, biocompatibility, low immunogenicity, and minimal or no toxicity (Herdiana et al., 2021). The positively charged (cationic) nature of Ct and NCt amplified their adhesion/attachment to negatively charged biological surfaces (e.g., microbial cells, cancer cells, and mucosal membranes) through electrostatic interactions, which provide significant rationale to these biopolymers for drug delivery and internalization into targeted cells/systems (Ehrbar et al., 2020; Oh et al., 2021). By *in vivo* enzymes, Ct and NCt normally break down into CO_2_ and H_2_O, guaranteeing adverse effects to somatic cells, and due to their elevated solubility in low acidic solutions (normally presented in the tumor microenvironment), NCt and Ct are frequently used in developing effective tumor-targeted composites (Ehrbar et al., 2020; Herdiana et al., 2021).

Curcumin (Cur) is the chief component of turmeric (*Curcuma longa*), which exhibits significant pharmacological and nutritional effects in humans; Cur biosafety for human and animal trials was confirmed, even with excessive doses (Mahmood et al., 2015). Cur is a hydrophobic molecule; its low bioavailability has always been the main problem for its biomedical applications, although this bioactive molecule proved its efficacy for managing neurological, cardiovascular, inflammatory, metabolic, liver and skin diseases, and numerous cancer types (Anand et al., 2008; Ravindran et al., 2009; Mahmood et al., 2015). The use of nanocarriers (e.g., nano-polymers like NCt), for Cur conjugation, loading, and delivery, could surprisingly overcome this difficulty and greatly increase its bioavailability/bioactivity as a powerful anticancerous nanocomposite (Chuah et al., 2014; Kunnumakkara et al., 2019; Almutairi et al., 2020; Hu and Luo, 2021).

The nanoconjugation of NCt/Cur was effectively achieved and provided augmented synergistic actions for inhibiting/killing diverse tumor cells, which recommended their clinical application as low-risk, biocompatible, and bioeffective composites (Li et al., 2020; Valizadeh et al., 2021; Yu et al., 2021).

Eugenol (Eug) is the 4-allyl-2-methoxyphenol fragrant oily compound that is predominantly acquired from clove oil; Eug is widely used for its outstanding rationales, e.g., powerful antioxidant, anti-inflammatory, antimicrobial, and antitumor actions (Ulanowska and Olas, 2021). Eug, with its multidirectional actions, is endorsed for application in numerous disciplines, including food preservation, antimicrobial drugs, and antitumor agents (Nisar et al., 2021). The Eug anticancer actions could involve diverse mechanisms, e.g., triggering cell death, arresting metastasis and the cell cycle, constraining cell migration and angiogenesis, and investigating numerous cancer lines (Li et al., 2020; Ulanowska and Olas, 2021); Eug-based formulations were additionally used as a potential adjunct remedy for post-treatment complications after conventional chemotherapy. Due to low Eug chemical stability, susceptibility to oxidation, and biochemical interactions, it was recommended to combine/load it with other biomolecules (e.g., biopolymers, nanopolymers, and polysaccharides), which led to enhanced effectiveness and stability with diminished toxicity (Zari et al., 2021). The NCt/Eug nanoconjugates were used efficaciously in preserving foods and genetically inhibiting aflatoxin B1 synthesis (Das et al., 2021). In cancer treatments, Eug encapsulation within nanopolymers such as NCt provided a successful approach for avoiding its prompt absorption by organs, enhancing their water solubility, and accordingly augmenting their combined action (Khalil et al., 2017; Zari et al., 2021).

The nanocomposites (NCs) involve the conjugation of many particles in their nanoforms, chemically, physically, or electrostatically. The polymeric NCs can provide numerous opportunities and usages, e.g., in tissue engineering, nano-carrying, disease theranostics, antimicrobials, drug delivery, and anticancer properties (Feldman, 2019). Great advantages were proved for polymer NCs (micelles, polymersomes, liposomes, and hydrogels) for cancer diagnosis/treatment due to their unique properties (e.g., eco-friendly nature, design capacity, cost-effectiveness, and facile production). The polymer-based NCs that respond to diverse stimuli can provide platforms for efficient drug deliveries with controlled rates and stable bioactivities (Feldman, 2019; Ehrbar et al., 2020; Herdiana et al., 2021).

No investigations were found regarding the combined loading of NCt with Cur and Eug to examine their synergism for inhibiting cancerous cells.

Therefore, the NCt synthesis and its loading with Cur and Eug were used here to evaluate their anticancer bioactivities and their synergistic actions toward the *in vitro* eradication of colorectal and breast adenocarcinoma cells.

## 2 Materials and methods

### 2.1 Chitosan extraction and characterization

The wasted shells of mantis shrimp (*Erugosquilla massavensis*) were used as sources for chitosan extraction. Mantis shrimps were harvested from the Egyptian Mediterranean Sea at Port Said Governorate; the shells were manually removed, and a total of 500 g were extensively cleansed, dried with 45°C hot air, and pulverized. The Ct extraction (Tayel et al., 2020) involves deproteinization [immersion in 28 folds (w/v) from 2.0 N NaOH solution, 24 h, 25°C]; demineralization [immersion in 28 folds (w/v) from 2.0 N HCl solution, 24 h, 25°C]; and deacetylation [immersion in 30 folds (w/v) from 55% NaOH solution, 5 h, 125°C]. Each of the previous steps was followed by extensive washing with deionized water (DW), drying at 45°C, and pulverization. The Ct molecular weight (MW) was gauged *via* high-performance GPC (gel permeation chromatography) (Terbojevich et al., 1993), while the deacetylation degree (DD) measurement depended on FTIR spectrum Ct (Fourier transform infrared spectroscopy, Perkin Elmer™, FTIR v10.03.08, Germany), from its absorbance ratio of A1655/A3450 (Tayel et al., 2016). The conditions for MW determination with high-performance GPC included the injection of 30 μl of the Ct solution (0.1%), eluted with 0.2 M sodium acetate/0.5 M acetic acid as a mobile phase, at a flow rate of 0.8 ml/min and 40°C, using an HPLC chromatographic station (Waters, Milford, MA) equipped with a Shodex OHpak column (8.0 × 300 mm; SB-805HQ, Showa Denko Co., Japan).

The structural/crystalline attributes of Ct were additionally traced with the X-ray diffractometer (Siemens, D500, Munich, Germany) using the following measurement conditions: Cu-Ka radiation (λ = 1.54 Å and 2θ angle changeable from 5° to 70°) with 4.8 min^−1^ scanning rate, 40 kV voltage, and 30 mÅ intensity.

### 2.2 Loading of nanochitosan with bioactive compounds

Based on preliminary trials (data not shown) and previous investigations (Agustinisari et al., 2020; Almutairi et al., 2020), the following concentrations were applied for fabricating the NCt-based nanoconjugates using the crosslinking emulsion/ionic-gelation protocol: Ct solution [1.0% (w/v) in 1.5% acetic acid aqueous solution]; Cur solution [1.0% (w/v) in 95% ethanol]; TPP [Na-tripolyphosphate 1.0% (w/v) in DW], while both Eug and Tween 80 were directly used for preparation. First, the Ct solution (100 ml) was amended with 1 ml of Tween 80 and speedily stirred for 120 min, and then, Cur solution (100 ml) or Eug (1 ml) or their composites was slowly pipetted into that solution by stirring (520 x g for 100 min). Then, 100 ml of TPP solution was subsequently dropped into these solutions (at a rate of 0.35 ml/min) while being stirred at 630 x g, and the stirring was continued for another 120 min after the TPP solution dropping. The formed NCt/Cur, NCt/Eug, and NCt/Cur/Eug nanoconjugates were harvested *via* centrifugation (20 min at 8,100 × *g* speed), washed with DW and 1.0% (v/v) Tween 80 solution (to remove non-nanoparticle components), re-centrifuged, and freeze-dried. For plain NCt preparation, neither Cur nor Eug were added.

### 2.3 Characterization of synthesized nanoconjugates

#### 2.3.1 FTIR spectral analysis

The FTIR analysis (Perkin Elmer, Germany) of used/synthesized materials (e.g., plain NCt, plain Cur, plain Eug, NCt/Cur, NCt/Eug, and NCt/Cur/Eug) was conducted in the 450–4,000 cm^−1^ wavenumber range using the FTIR transmission mode after samples’ amalgamation with KBr.

#### 2.3.2 Structural analysis

The structures and features of synthesized nanoconjugates (NCt/Cur, NCt/Eug, and NCt/Cur/Eug), e.g., distribution, shape, and size, were assessed; the Zetasizer photon correlation spectroscopy (PCS Zetasizer, Malvern™, United Kingdom) was used to assess the nanoconjugates’ charge and particle size (Ps) distribution, whereas the SEM (scanning electron microscopy, JEOL JSM-6301F, Tokyo, Japan) imaging emphasized the nanoconjugates’ morphology, apparent size, and particle distribution. The magnification of ×15,000 was applied at an acceleration of 10 kV. The elemental composition of NCt was assessed *via* coupled energy-dispersive X-ray (EDX) spectroscopy on the SEM instrument.

#### 2.3.3 Nanochitosan entrapment efficiency and loading capacity

The entrapment efficiencies (EE %) and loading capacities (LC %) of Cur and Eug within NCt particles were determined by pelletizing the loaded samples at 18,500 x *g* for 22 min. The resulting pellets were re-dispersed and lyophilized. A total of 2 mg from each lyophilized sample were dispersed in ethanol (10 ml) and sonicated for 7 min, and the dispersion solutions were centrifuged as mentioned earlier. The supernatants were collected after centrifugation, and the biomolecules’ (Cur and Eug) amounts within these supernatants were spectrophotometrically quantified (using SHIMADZU, UV-2450, Japan) at maximum absorptions of 429 and 284 nm for Cur and Eug, respectively (Anitha et al., 2011; Mondéjar-López et al., 2022). The EE (%) was based on ratios of existing biomolecules’ amounts in NCt to the amount of biomolecules used for loading process.

$$EE\;\left(\%\right)=\left[Total\;amount\;of\;biomolecules\;within\;the\;pellet/Initial\;taken\;amount\;of\;biomolecules\;for\;loading\right]\times 100.$$

Additionally, the LC (%) of biomolecules was calculated pertaining to the yield of obtained nanoparticles after centrifuging.

$$LC\;\left(\%\right)=\left[Total\;amount\;of\;entrapped\;biomolecules\;within\;the\;pellet/Yield\;of\;biomolecule-loaded\;NCt\right]\times 100.$$

#### 2.3.4 Biomolecules’ release assessment

The releases of biomolecules (Cur and Eug) from capsulated NCt were assessed under simulated conditions of gastric fluid (SGF; pH 1.2, using HCl and NaCl mixtures) and colon fluid (SCF; pH 7.2, using the phosphate saline buffer) throughout a duration of 60 h. The SGF and SCF were prepared as previously described (Han et al., 2020), involving the addition of 0.5% from HCl and NaCl to DW and adjusting the pH to 1.2 with 0.1 M NaOH/HCl solutions for SGF preparation. For SCF preparation, 0.05 M potassium dihydrogen phosphate in DW was prepared, and the pH was adjusted to 7.2. The *in vitro* releases were conducted in a dissolutor instrument (M. 299-6TS; Ethik Technology, Brazil), where definite amounts of biomolecule-loaded NCt were dispersed in distinct SGF and SCF aqueous solutions (Han et al., 2020). Using constant conditions of 52 x *g* and 37°C, the biomolecules’ releases were tracked after periodic intervals, where 1.1 ml of each release solution was collected for evaluation and replaced with fresh buffers. The cumulative biomolecules’ release percentages were measured *via* a UV spectrophotometer, as indicated earlier. The biomolecules’ release was calculated as a percentage of the initial loaded amounts at the beginning of the experiments.

### 2.4 Anticancer assessment

#### 2.4.1 Cancerous cell culture

The screened cancer cell lines, e.g., CaCo2 (colorectal adenocarcinoma) and MCF7 (breast adenocarcinoma), were obtained from the VACSERA-EG (Egyptian Company for Vaccine and Serum, Cairo, Egypt). Reagents for cell culture [D-MEM (Dulbecco modified Medium), FBS (fetal bovine serum), trypsin, l-glutamine, streptomycin/penicillin, DMSO (dimethyl sulfoxide), PSB (phosphate saline buffer), and MTT (3-(4,5-dimethylthiazol-2-yl)-2,5-diphenyltetr-azolium bromide)] were provided by GIBCO (Brooklyn, NY). Cells were cultured/maintained in supplemented D-MEM with FBS (10%), l-glutamine (2%), and penicillin/streptomycin (1%) in a humidified atmosphere (5% CO_2_) at 37°C. Cells were rinsed periodically with PSB (pH 7.4), detached with trypsin–EDTA (0.25%), and re-cultured in fresh supplemented D-MEM, usually every 48 h, after cells reached ∼80–90% confluence. The normal cells (e.g., L929 parent cell line) were used for comparison.

#### 2.4.2 MTT cytotoxicity assay

CaCo2 and MCF7 cells were individually seeded in microtiter plates (96 wells, flat bottom) containing supplemented D-MEM (100 μl/well, ∼1×10^4^ cells) and incubated for 24 h, as in the previous conditions. Gradual concentrations (e.g., 0, 1.56, 3.125, 6.25, 12.50, 25.0, 50.0, and 100.0 μg/ml) of individual composites (e.g., NCt/Cur, NCt/Eug, and NCt/Cur/Eug) were applied onto cell-containing wells and incubated for 24 h. Subsequently, MTT (5.0 mg/ml) was appended to wells, and plates were incubated for another 4 h. The medium was then detached, and 100 μL/well of DMSO was appended and vortexed mildly for 22 min. The colorimetric absorbance (at 570 nm) was measured and compared with the standard curve. Bare biomolecules (e.g., NCt, Cur, and Eug) were screened at the same conditions for comparison.

#### 2.4.3 Comet “single-cell gel electrophoresis” assay

The purpose of the comet assay was to identify the DNA fragmentation in cancerous cells after treatments with the nanocomposites (Badawy et al., 2018). The cells’ DNA was stained using GelRed, which provides a reddish color in fluorescent microscope examination. The migration patterns of DNA fragments from cancerous cells (100 cells/trial) were evaluated through the ×40 objective lens of the fluorescence microscope (OLYMPUS-IX71, Japan) with an excitation filter of 420–490 nm and an issue of 510 nm. In 24-well microplates, the CaCo2 cells were exposed to PSB (the negative control) or the nanocomposites (NCt/Cur, NCt/Eug, and NCt/Cur/Eug) at their IC_50_ values for 24 h. Treated cells were rinsed with PSB twice, trypsinized, centrifuged (at 182 × g for 5 min), resuspended in 0.6% agarose (low melting point), and then dropped onto previously coated slides with standard 1% agarose. After solidification in the freezer for 12 min, slides were positioned in lysis buffer (containing 10 mM Tris, 1% Triton X-100, 10% DMSO, 2.5 M NaCl, and 100 mM EDTA with pH 10) and incubated at 4°C overnight. The electrophoresis was subsequently performed for 18 min under regular conditions (300 mA; 25 V; 1.25 V/cm). Finally, the slides were stained for 3 min with a GelRed solution (1:10.000 v/v) and subjected to fluorescence microscope analysis. The parameters of DNA damage (including the tail length of degraded DNA, the tail moment, and the degraded DNA ratio) were assessed using KOMET-V software (Kinetic Imaging Inc., Liverpool, United Kingdom).

### 2.5 Statistical analysis

The results were analyzed by the “one-way ANOVA” test (GraphPad Prism 5, La Jolla, CA) for determining group differences. The differences between the results’ means ± SE (standard errors) were calculated by Tukey’s HSD “Tukey’s honestly significant difference”, and the significances were set at *p* < 0.05.

## 3 Results and discussion

### 3.1 Chitosan extraction

The Ct was promisingly extracted from *E. massavensis* shells with a yield of 25.81%; the produced powder had a light color (creamy-white), a MW of 42.6 kDa, and a DD of 90.7%. The obtained DD (≥70%) and MW of the produced Ct validated the mantis’s chitin transformation to Ct with low MW (Rinaudo, 2006). The obtained physicochemical characteristics of produced Ct are in accordance with those previously reported for the extracted and commercial (standard) Ct types (Rinaudo, 2006; Ben Seghir and Benhamza, 2017; Zhao et al., 2018; Jesus et al., 2020; Hu and Luo, 2021); they could authorize that the obtained high DD and low MW for extracted Ct in the current study are good indicators for its extraction and attributes. The XRD analysis of the produced Ct (Supplementary Data, Supplementary Figure 1S) illustrated that the product had low crystallinity attributes, mainly due to its intra-molecular H-bonds resulting from chitin deacetylation (Ben Seghir and Benhamza, 2017).

The crystallinity degree (CrI) was calculated to appraise the Ct purity; the attained value is approximately equivalent to 71.2%. This reduced value is principally attributed to subsequent intermolecular bonding of hydrogen after deacetylation, and it indicates the amorphous state of the Ct molecular structure (Ben Seghir and Benhamza, 2017).

### 3.2 FTIR analysis

**FIGURE 1:**
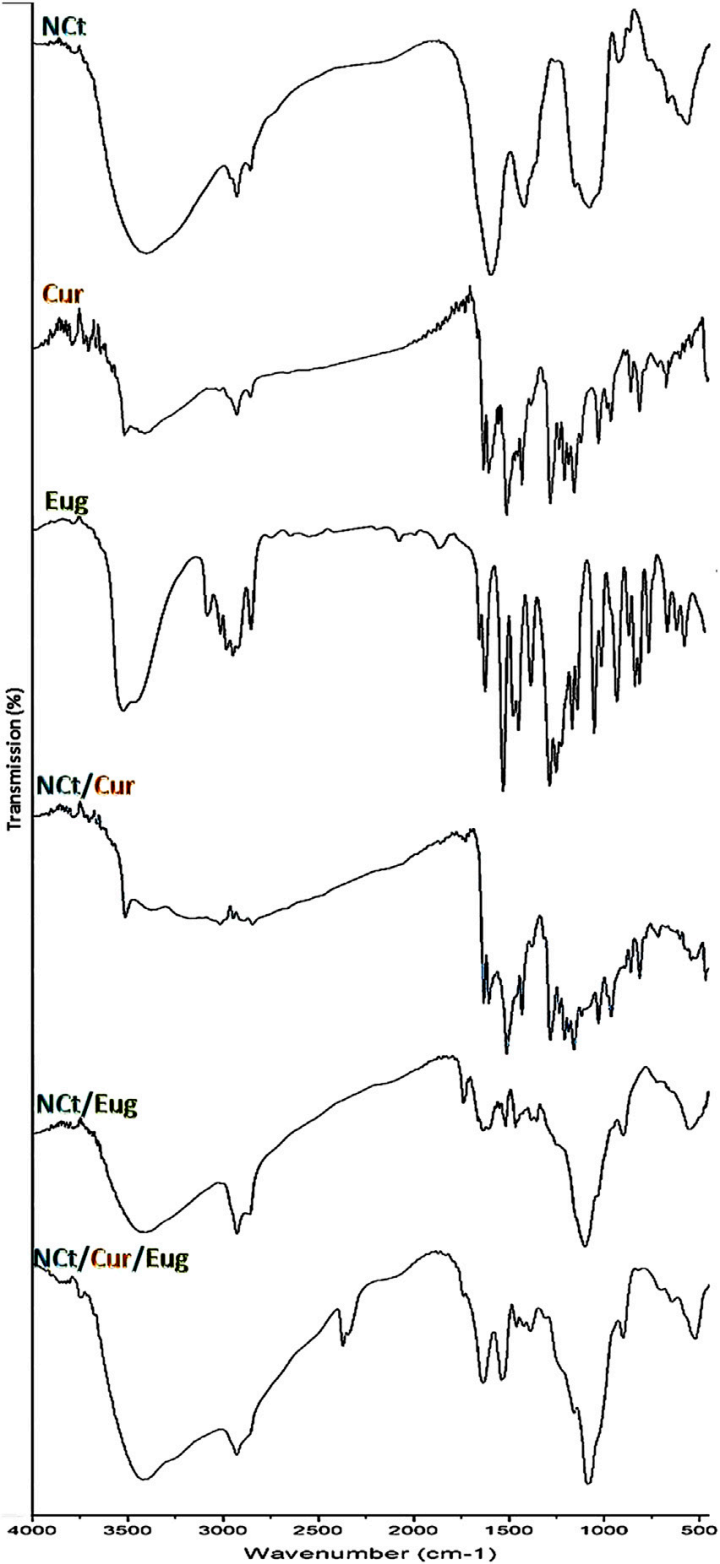
FTIR spectra of used agents/composites, including chitosan nanoparticles (NCt), curcumin (Cur), and eugenol (Eug).

The infrared (IR) analyses, of used/synthesized agents and nanocomposites in the current study, are presented in Figure 1 to emphasize their potential chemical structures and interactions. The analysis of NCt IR spectrum (Figure 1-NCt) indicated that NCt had the main distinctive features of native chitosan (Feldman, 2019; Alalawy et al., 2020); the principal NCt characteristic peaks were detected at 654 cm^−1^ (for ‒OH vibrations), 1,031 cm^−1^ (for C‒O stretching), 1,420 cm^−1^ (for –CH_2_ vibrations), 1,564 cm^−1^ (appointing N‒H bends in primary amines), 1,647 cm^−1^ (appointing C=O stretching in secondary amides), and 3,414 cm^−1^ (wide band indicating ‒H intermolecular bonds) (Divya and Jisha, 2018; El Rabey et al., 2019). The NCt IR spectrum also appointed the C=O symmetric/asymmetric vibrations in secondary and tertiary amides at 1,327 cm^−1^ (Tayel et al., 2021).

The Cur spectrum (Figure 1-Cur) emphasized its distinctive characteristics, which were evidenced through the peaks at 3,509 cm^−1^ (O‒H stretching vibration in phenolics), 1,618 cm^−1^ (C=C stretching in aromatic moiety), 1,502 cm^−1^ (C=C and C=O vibrations), 1,415 cm^−1^ (C‒H bending vibrations in olefinics), 1,251 cm−1 (C‒O stretched vibrations in aromatics), and 1,021 cm^−1^ (C–O–C stretched vibrations) (Khan et al., 2016; Almutairi et al., 2020). The IR peaks of Cur at 732 cm^−1^, 810 cm^−1^, and 951 cm^−1^ appointed the vibrated bending –CH in alkene groups (Ilyas et al., 2018).

The IR analysis of Eug (Figure 1-Eug) indicated the −OH groups’ presence in carboxylic acids at ∼3,450–3,550 cm^−1^; the band at 3,097 cm^−1^ indicated the C−H bonds, whereas the bands in the 1,475–1,625 cm^−1^ range indicated the C=C vibrations in aromatic rings, and the bands in the 800–900 cm^−1^ range specified the aromatic substitution. Additionally, the acid carbonyl bonds/groups are designated with bands around 1,725 cm^−1^, whereas the methoxy group (−O−CH_3_) was indicated at ∼1,035 cm^−1^ (Siddique et al., 2018; Djunaidi et al., 2019). The IR peaks at 1,634, 996, and 917 cm^−1^ could appoint the C=C in vinyl groups, and the sharp peaks at 1,634, 1,613, and 1,517 cm^−1^ can also appoint the stretched C=C in the aromatic moiety (Pramod et al., 2015; Siddique et al., 2018).

The IR spectrum of Cur/NCt nanoconjugates (Figure 1-NCt/Cur) displayed several distinctive peaks relevant to their original sources from NCt or Cur spectra, which could imply the cross-linkages (covalently and electrostatically) amongst the compounds’ molecules (Rejinold et al., 2011; Almutairi et al., 2020); the chemical interactions between the NCt amine groups and Cur keto groups were suggested as the key principal for loading Cur as a drug onto NCt (Khan et al., 2016; Vaiserman et al., 2020). The Cur designative groups could be identified in the Cur/NCt conjugated spectrum.

In the Eug/NCt spectrum (Figure 1-NCt/Eug), many Eug designative groups (within the 1,330–1,520 cm^−1^ range) could be identified in the Eug/NCt nanoconjugates. The apparent peaks at 608, 1,509, and around 1,635 cm^−1^ were comparable to those in the Eug spectrum. The main NCt characteristic IR peaks were evidently detected in the matrix. The O‒H and N‒H indicative peaks in NCt (around 3,400 cm^−1^) were overlapped with the Eug peak in their mixture (Pramod et al., 2015).

Additionally, the Eug characteristic peaks for ‒CH_3_ bending (at 1,425–1,460 cm^−1^) were detected in NCt/Eug conjugates, and many peaks were slightly shifted, which indicate the potential interactions between the two molecules (Cahyono et al., 2018).

The combined matrix spectrum from the entire molecules (NCt, Cur, and Eug) exhibited the distinctively characterized peaks from each agent (Figure 1-NCt/Cur/Eug), which strongly advocated the occurrence of chemical interactions within the matrix molecules. The covalent/electrostatic interactions between NCt, Cur, and Eug could be presumed to augment their combined bioactivities and actions (Almutairi et al., 2020; Vaiserman et al., 2020).

### 3.3 Structural analysis of nanocomposites

The fabricated NPs and nanocomposites (e.g., NCt, NCt/Cur, NCt/Eug, and NCt/Cur/Eug) had reasonable particle sizes (Ps) and zeta potentialities (Table 1). The mean Ps of NCt (268.5 nm) was less than their Ps’ means after conjugation with Cur and Eug (314.9 and 296.4 nm, respectively). The largest Ps mean was recorded after loading NCt with both Cur and Eug (364.7 nm). The used concentrations and fabrication procedure were effective for generating tiny Ps and elevated surface charges, compared with recent investigations for preparing parallel nanoconjugates (Agustinisari et al., 2020; Almutairi et al., 2020; Anand et al., 2021).

**TABLE 1:** Size distribution and zeta potentials of fabricated nanoparticles^a^.

Nanomaterial	Size range (nm)	Mean diameter (nm)	Zeta potential (mV)	PDI ± SD
NCt	106.2–543.9	268.5	+37.5	0.491 ± 0.05
NCt/Cur	197.6–662.8	314.9	+32.8	0.628 ± 0.08
NCt/Eug	149.8–583.6	296.4	+31.7	0.606 ± 0.07
NCt/Cur/Eug	211.2–694.4	364.7	+29.4	0.643 ± 0.10

^a^ NCt: chitosan nanoparticles, Cur: curcumin, and Eug: eugenol.

The synthesized nanoconjugates had relatively high positive charges on their surfaces (e.g., +37.5, +32.8, +31.7, and +29.4 mV for NCt, NCt/Cur, NCt/Eug, and NCt/Cur/Eug, respectively); these charges, especially in NCt, were proposed to arise from amino group protonation in NCt molecules. Thus, the decrements in NCt surface charges after conjugation with Cur and Eug were expected *via* charge shielding and the occupation/coating of these biomolecules within NCt molecules; the involvement of more loaded molecules (drugs) or their elevated concentrations in NCt was also reported to reduce the composite zeta values (Keawchaoon and Yoksan, 2011; Agustinisari et al., 2020). However, the attained high charges on nanocomposite surfaces (∼+30 mV) are assumed to augment the NP stability, availability, and delivery to targeted tissues (Sahoo et al., 2007). The representative zeta analysis curves for NCt/Cur, NCt/Eug, and NCt/Cur/Eug are provided in Supplementary Data (Supplementary Figure 2S). The PDI value is one of the important indicators of nanoparticles’ dispersion homogeneity and the system stability (Jesus et al., 2020; Ahmad et al., 2021; Oh et al., 2021). The PDI measurements are represented as numerical values (ranging from 0.0 to 1.0), where lower PDI values indicate higher stability. Accordingly, the attained PDI values for synthesized nanoparticles/composites here indicate the particle tendency toward polydispersion (Keawchaoon and Yoksan, 2011; Woranuch and Yoksan, 2013; Anand et al., 2021).

**FIGURE 2:**
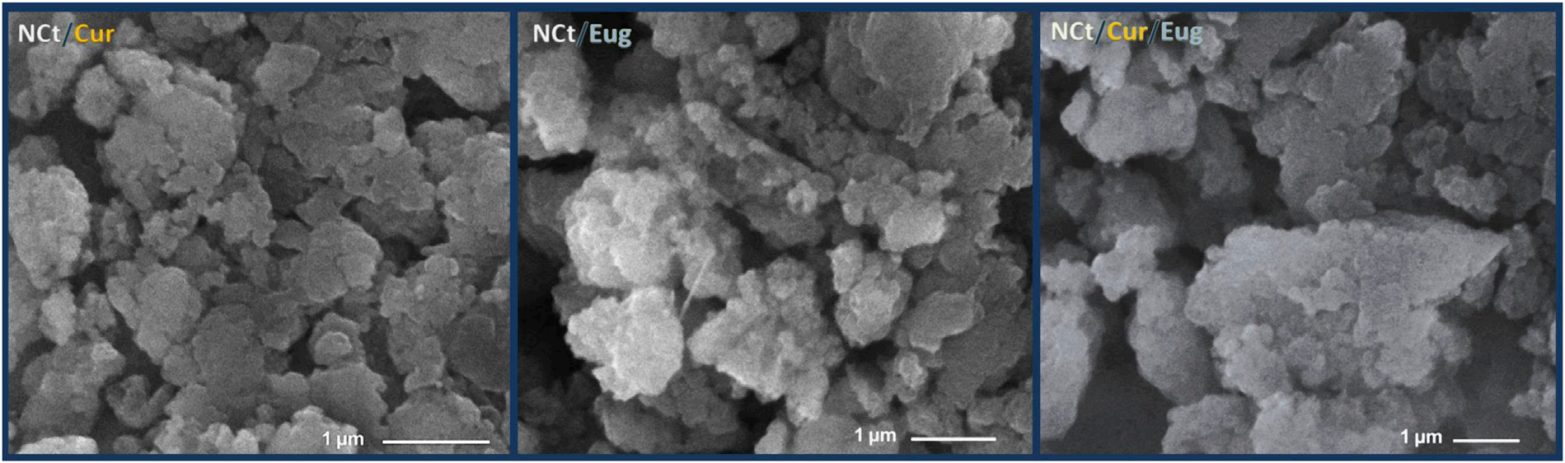
Scanning ultrastructures of synthesized nanocomposites from chitosan nanoparticles (NCt), curcumin (Cur), and eugenol (Eug).

The SEM photographs of synthesized nanocomposites (NCt/Cur, NCt/Eug, and NCt/Cur/Eug) indicated their structures and size distributions (Figure 2). The conjugated NPs appeared with homogenous round (spherical) shapes and average estimated diameter means of 321.2, 305.7, and 373.9 nm for NCt/Cur, NCt/Eug, and NCt/Cur/Eug, respectively, as computed by the SEM instrument. The computed Ps means of the conjugates’ particles were in parallel with the DLS results, as illustrated in Table 1.

The observed EDX spectra in Supplementary Data (Supplementary Figure 3S) for synthesized NCt represent the elemental composition of the nanoparticles, which included elements such as carbon, nitrogen, oxygen, and phosphorus. This analysis validated the interaction of TPP with the native Ct and verified the NCt synthesis (Divya and Jisha, 2018; Yu et al., 2021; Mondéjar-López et al., 2022).

### 3.4 Loading capacity and release of biomolecules from NCt

The EEs of Cur and Eug within NCt were estimated to be 81.3 ± 1.4% and 17.5 ± 0.7%, whereas the LCs of the biomolecules within NCt were 14.3 ± 0.6 and 10.7 ± 0.7, respectively.

**FIGURE 3:**
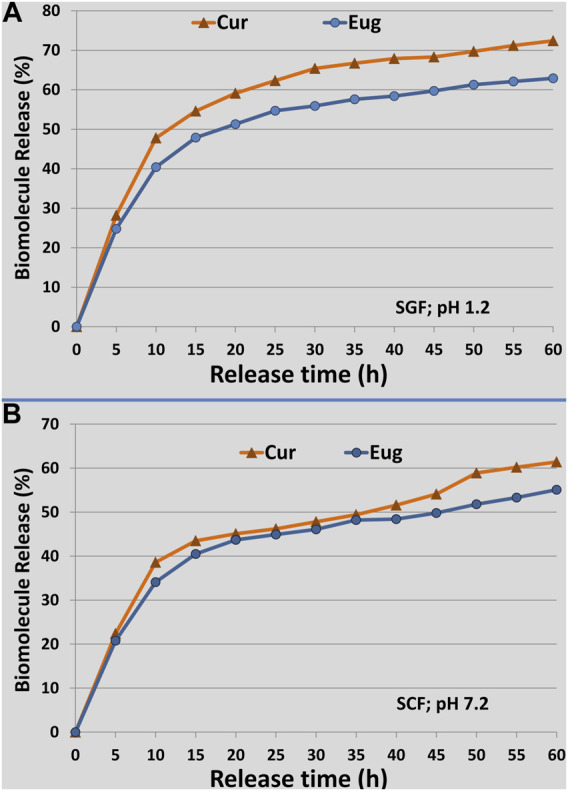
Release patterns of loaded curcumin (Cur) and eugenol (Eug) into chitosan nanoparticles under simulated conditions of gastric fluid [**(A)**; SGF; pH 1.2] and colon fluid [**(B)**SCF; pH 7.2], throughout the release duration of 60 h.

The release patterns of Cur and Eug from NCt particles under the SCF and SGF conditions (pH 7.2 and 1.2, respectively) are indicated in Figure 3. The biomolecules’ releases were evidenced as pH- and time-dependent; the released amounts of Eug and Cur from NCt were higher in SGF than in SCF conditions. The patterns designated burst biomolecule releases within the first 10 h followed by gradual slow releases over experiment continuation for 60 h (Figure 3). The entrapped Cur had higher release patterns than Eug from NCt, under both SGF and SCF conditions, with maximum Cur releases of 72.4 and 61.2%, respectively, whereas the maximum releases of Eug from NCt after 60 h were 62.9 and 55.1% within SGF and SCF conditions, respectively.

The biomolecules (Cur and Eug) that are adsorbed onto the NCt surface or entrapped nearby these surfaces could be responsible for the burst initial releases because the NCt and biopolymers’ dissolution rates are regularly high near their surfaces (Anitha et al., 2011; Almutairi et al., 2020); thus, the released amounts of loaded biomolecules will be high too. The Cur and Eug releases were faster in SGF (acidic pH) than in SCF (neutral pH); this can be explained by the prompt swelling behavior of NCt in acidic environments due to the protonation of its amine groups in such conditions, which leads to faster molecule releases at that low pH (Anitha et al., 2011). No contradiction is found between the LC and release patterns of biomolecules from NCt, as the release was calculated as a percentage of the entrapped amounts in the nanoparticles. Tumors frequently have poor architectures of blood vessels, which result in metabolic accumulation in their microenvironment with elevated acidic pH (Han et al., 2020). Therefore, the faster releases of bioactive molecules from NCt under acidic conditions could have potential importance for application in cancer treatments (Almutairi et al., 2020; Hu and Luo, 2021).

### 3.5 Cytotoxicity assessment of nanocomposites

**FIGURE 4:**
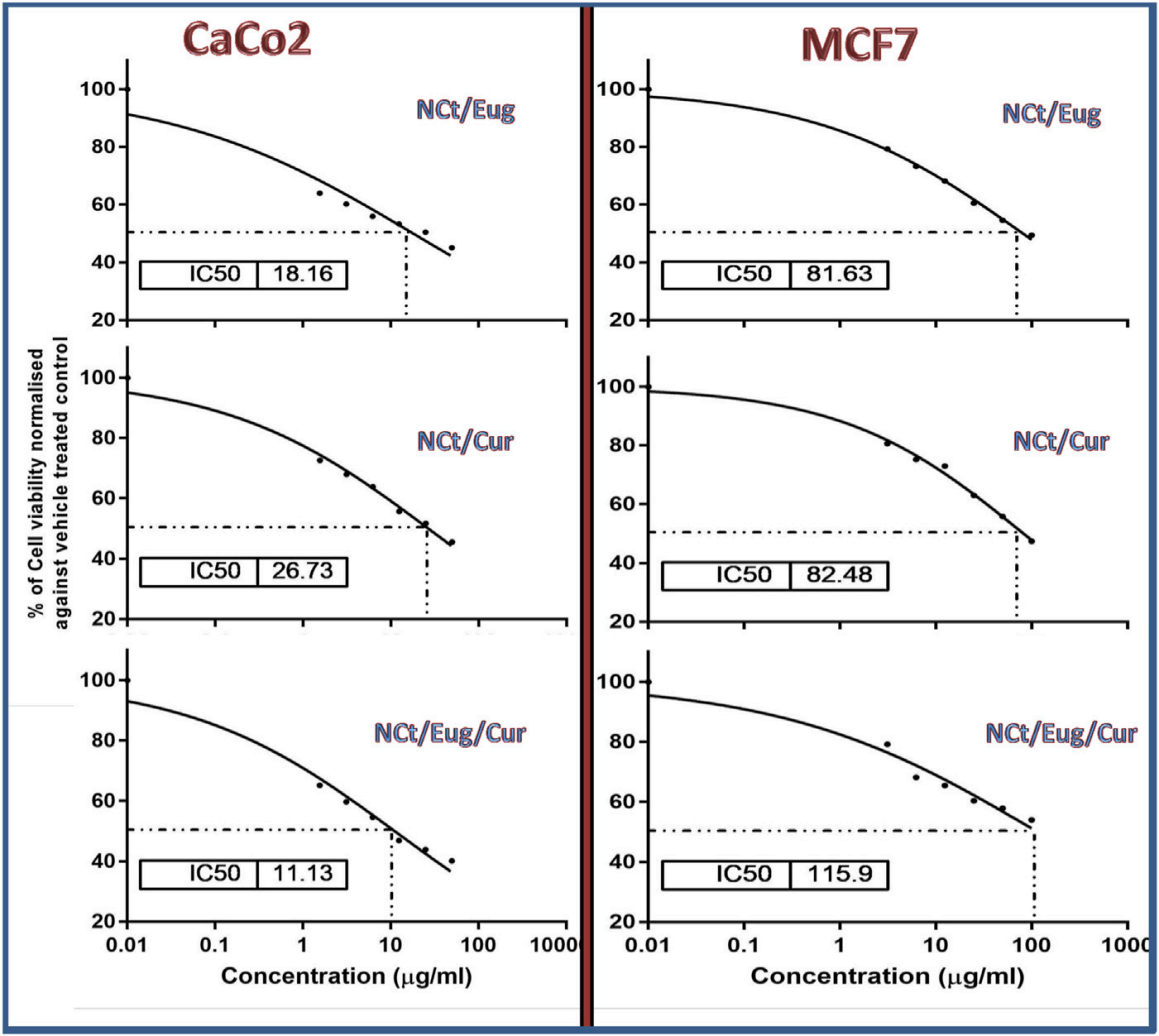
Cytotoxicity of formulated nanocomposites from nanochitosan (NCt), eugenol (Eug), and curcumin (Cur) toward CaCo2 and MCF7 cancerous cells using the MTT assay.

The cytotoxic impacts of nanocomposites (NCt/Eug, NCt/Cur, and NCt/Eug/Cur) toward CaCo2 and MCF7 cancerous cells, using the MTT assay, are illustrated in Figure 4. The results displayed significant elevated anti-proliferative and dose-dependent activities of all nanocomposites against treated cells. The combined nanocomposites (NCt/Eug/Cur) emphasized the highest significant activity against CaCo2 cells, followed by Cur/Eug and NCt/Cur. For MCF7 cells, the most effective composite was NCt/Eug, followed by NCt/Cur and NCt/Eug/Cur nanocomposites. Generally, CaCo2 cells were significantly more sensitive to nanocomposites than MCF7 cells, as evidenced by their IC_50_. The negative controls showed no significant detectable activities toward cells. The least significant IC_50_ (11.13 μg/ml) was obtained after CaCo2 treatment with the NCt/Eug/Cur nanocomposite. The plain biomolecules (NCt, Cur, and Eug) showed significantly lower toxicities than the nanocomposites (data not shown). All biomolecules (i.e., bare NCt, Cur, Eug, and the formulated nanocomposites) exhibited minimal cytotoxicities toward the parent L929 normal cell line derived from subcutaneous adipose areolar tissues.

The nanocomposition of NCt with examined bioactive compounds resulted in elevated cytotoxicity levels toward cancerous cells; this was formerly demonstrated with the administration of NCt/Cur for inhibiting HT-29 and other types of cancerous cells (Chuah et al., 2014; Hu and Luo, 2021) and for the NCt/Eug nanocomposites toward melanoma (A-375) and cancerous breast cells (MDA-MB-468) (Valizadeh et al., 2021).

Although the plain NCt does not regularly exhibit detectable cytotoxicity toward cells (Chuah et al., 2014), it can provide great enforcement and augmentation to the anticancer bioactivities/cytotoxicities of natural products and drugs when NCt was used as a carrying/delivering agent for these compounds (Almutairi et al., 2020; Detsi et al., 2020; Mazzotta et al., 2020; Anand et al., 2021). NCt was stated as the ideal biopolymeric system for delivering candidate anticancer drugs (Ahmad et al., 2021); the NCt’s positive charges and adsorption capacities facilitate its attachment to cancerous cells and delivering the anticancer compound into the cells (Detsi et al., 2020; Mazzotta et al., 2020; Ahmad et al., 2021). Promisingly, the conjugation of both Cur and Eug in the NCt delivery system resulted in powerful synergistic action against CaCo2 cells, but the contrary happened toward MCF7 cells; this could confirm the diverse natures of cancerous cells toward specific agents/composites because each cell type has its own physiological and enzymatic characteristics that influence their susceptibility to anticancer compounds (Adhikari and Yadav, 2018; Zari et al., 2021).

### 3.6 Consequences of nanocomposite exposure on DNA fragmentation of CaCo2 cells

**FIGURE 5:**
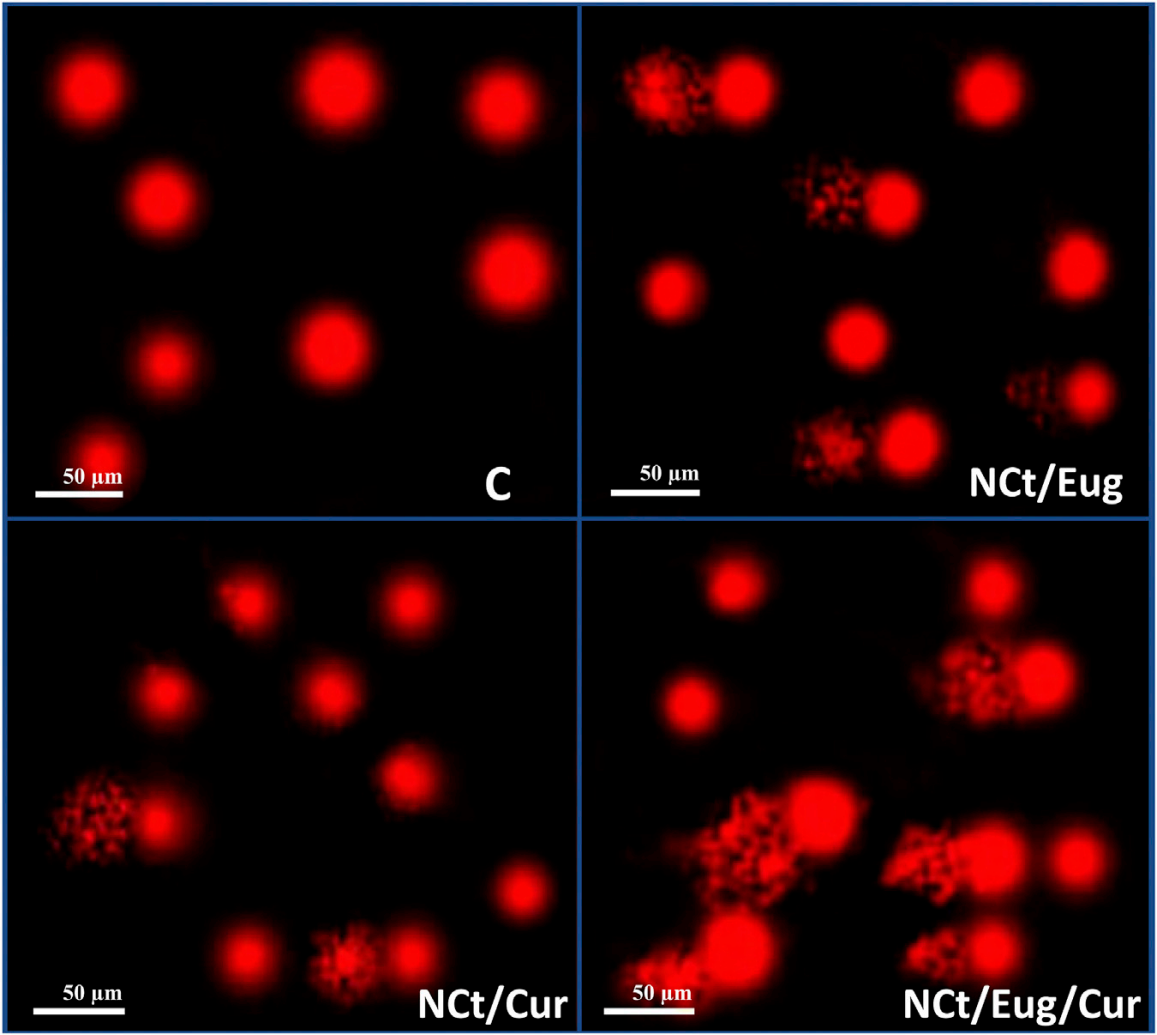
Comet images demonstrating CaCo2 cells with DNA fragmentation after treatment with IC_50_ from nanochitosan/eugenol (NCt/Eug), nanochitosan/curcumin (NCt/Cur), and nanochitosan/eugenol/curcumin (NCt/Eug/Cur) composites, compared to control (C) cells.

The consequences of CaCo2 cells’ exposure to IC_50_ from nanocomposites (NCt/Eug, NCt/Cur, and NCt/Eug/Cur), on the DNA fragmentation in cancerous cells, are represented in Figure 5 and Table 2. The considerable fragmentation in CaCo2 DNA was evidenced from comet images (Figure 5); all nanocomposites were able to induce detectable DNA damages and fragmentation in exposed cancerous cells. The effect of the NCt/Eug/Cur nanocomposite was the most powerful. The digital estimation of DNA fragmentation parameters indicated that all nanocomposites could significantly increase DNA damage in CaCo2 cells compared with the control (Table 2). Each of tailed DNA (%), tail moment, tail length, and tail DNA (%) significantly increases after cell treatment with NCt/Eug/Cur, NCt/Eug, and NCt/Cur, respectively. The longest tail length (9.54 ± 0.46 µm) was detected after cell treatment with NCt/Eug/Cur, compared with lower tail lengths from other treatments and the control group (Table 2).

**TABLE 2:** Software analysis of comet images for treated CaCo2 cells with IC_50_ from nanochitosan/eugenol (NCt/Eug), nanochitosan/curcumin (NCt/Cur), and nanochitosan/eugenol/curcumin (NCt/Eug/Cur) composites, compared to control (untreated) cells.

Group	Tailed %	Untailed %	Tail length (µm)	Tail DNA %	Tail moment
Control	2	98	1.68 ± 0.12^d^	1.54	2.59
NCt/Eug	19	81	7.33 ± 0.35^b^	5.60	41.05
NCt/Cur	16	84	6.09 ± 0.28^c^	4.78	29.11
NCt//Eug/Cur	30	70	9.54 ± 0.46^a^	8.17	77.94

The NCt served as a promising anticancer strengthening agent, especially with its loading with other bioactive molecules. The proposed mechanisms of NCt antitumor potentialities are associated with its ability to disturb the cell cycle and regular functions, restrict the central dogma of the biological system (e.g., RNA, DNA, enzymes, or proteins) synthesis, and disrupt the biosynthesis hormonal path, leading to the growth inhibition of cancerous cells (Adhikari and Yadav, 2018).

The powerful anticancer actions of the NCt/Cur nanocomposite indicate the elevated Cur bioavailability after loading into NCt, which empowered and augmented their combined antitumor activities and overcame the low availability of plain Cur (Kunnumakkara et al., 2019; Almutairi et al., 2020). Cur was among the highly advised phytocompounds to prompt the death of cancerous cells, although its exact molecular mechanisms are still somewhat unspecified; studies suggested Cur’s ability to restrain cancerous cells *via* affecting numerous crucial pathways signaling the cells’ angiogenesis, inflammation, apoptosis, proliferation, and survival (Ravindran et al., 2009). Cur could additionally influence many molecular cancer pathway regulators that are responsible for cell survival, proliferation, tumor suppressors, caspase activation, protein kinase, mitochondrial pathways, and death receptors (Anand et al., 2008; Kunnumakkara et al., 2019). The ROS (reactive oxygen species) production was also supposed to be a potential antitumor mechanism with the treatment of Cur; the efficacy of this biomolecule was remarkable even with treatments at low concentrations (Ravindran et al., 2009; Liu et al., 2017). The apoptosis induction in exposed cells was also the suggested mechanism of Cur to kill tumor lines (Zuo et al., 2021).

Regarding Eug, it was evidently supposed to trigger tumor cell apoptosis *via* diverse processes including ROS production and decrement of mitochondrial membrane potentiality, which gave it the apoptosis-triggering capability (Yoo et al., 2005; Zari et al., 2021). The Eug selective-targeting anticancer potentialities were documented for diverse cell lines and reported to be potentially strengthened after conjugation with further anticancer agents (Petrocelli et al., 2021; Zari et al., 2021); they additionally reported the need for an effective agent/system to deliver these actions to targeted cells. However, the NCt here could perform this delivery in very promising ways, either for Cur or Eug or their composites.

The key consequences of Eug as a forceful anticancer agent are cell necrosis induction, cell cycle arrest, and enforcing cell death. Eug could synergistically boost pro-apoptotic and cytotoxic actions (against colon cancers); suppress cell migration and boost apoptosis (in cervical cancers); upregulate the angiogenic and proinvasive factors, arrest cell development, and favor mitochondrial-initiated apoptosis (in gastric cancers); constrain DNA synthesis, stop cell cycle, and trigger apoptosis (in melanoma cells); and inhibit cancer-related oncogenes and antiapoptotic downstreams (in breast cancers) (Nisar et al., 2021; Petrocelli et al., 2021; Zari et al., 2021).

The anticancerous synergisms between Eug and other bioactive molecules (e.g., cinnamaldehyde, doxorubicin, and cisplatin) were documented toward various cancerous lines (Petrocelli et al., 2021; Zari et al., 2021); the current significant synergism between Eug and Cur in the NCt matrix and their elevated anticancer powers, especially against CaCo2 cells, could support these former reports. A graphical abstract illustrating the synthesis method and potential anticancer mechanism of green synthesized nanocomposites is provided in Supplementary Data (Supplementary Figure 4S).

## 4 Conclusion

Toward exploration of potential natural anticancer alternatives, NCt, Cur, and Eug were nanocomposited and evaluated against CaCo2 (colorectal adenocarcinoma) and MCF7 (breast adenocarcinoma) cells. The successful loading of both Cur and Eug into NCt was validated to provide effective anticancer nanocomposites that could eradicate CaCo2 and MCF7 cells. The innovative combination of the NCt/Cur/Eug nanocomposite was the most effective, especially for combating colorectal adenocarcinoma cells, which gives it more applicability for use in the oral route as a biosafe, natural, biocompatible, and powerful anticancer composite. This suggests the usefulness and effectiveness of natural molecules and their nanocomposites as promising anticancer agents and designates the synergistic effectiveness of conjugating NCt, Cur, and Eug in combined nanocomposites. Further studies for assessing the nanocomposites’ impact on cancerous cell membrane integrity using additional techniques (SEM, DNA degradation assay, protein degradation, and ROS production) are suggested as prospective investigations.

## Data Availability

The original contributions presented in the study are included in the article/Supplementary Material; further inquiries can be directed to the corresponding authors.

## Abbreviations

Ct, chitosan; NCt, chitosan nanoparticles; CaCo2, colorectal adenocarcinoma cells; GPC, gel permeation chromatography; Cur, curcumin; DD, deacetylation degree; DW, deionized water; D-MEM, Dulbecco modified Eagle medium; Egypt VACSERA-EG, Egyptian Company for Vaccine and Serum; EE, entrapment efficiency; Eug, eugenol; FBS, fetal bovine serum; FTIR, Fourier transform infrared spectroscopy; IC_50,_ half-maximal inhibitory concentration; LC, loading capacity; MTT, 3-(4,5-dimethylthiazol-2-yl)-2,5-diphenyltetr-azolium bromide; MCF7, breast adenocarcinoma cells; MW, molecular weight; NCs, nanocomposites; NPs, nanoparticles; TPP, Na-tripolyphosphate; L929, normal human cells; PSB, phosphate saline buffer; SEM, scanning electron microscopy; SCF, simulated colon fluid; SGF, simulated gastric fluid; Comet, single-cell gel electrophoresis; XRD, X-ray diffraction.
