# Supplementary material for: Fabrication and assessment of potent anticancer nanoconjugates from chitosan nanoparticles, curcumin, and eugenol
Source: Front Bioeng Biotechnol. 2022 Dec 8;10:1030936. doi: 10.3389/fbioe.2022.1030936 (PMC9773392; doi:10.3389/fbioe.2022.1030936)
Supplement: Supplementary file 1 [file DataSheet1.PDF]

Supplementary Data

Fabrication and Assessment of Potent Anticancer Nanoconjugates from Chitosan Nanoparticles, Curcumin and Eugenol

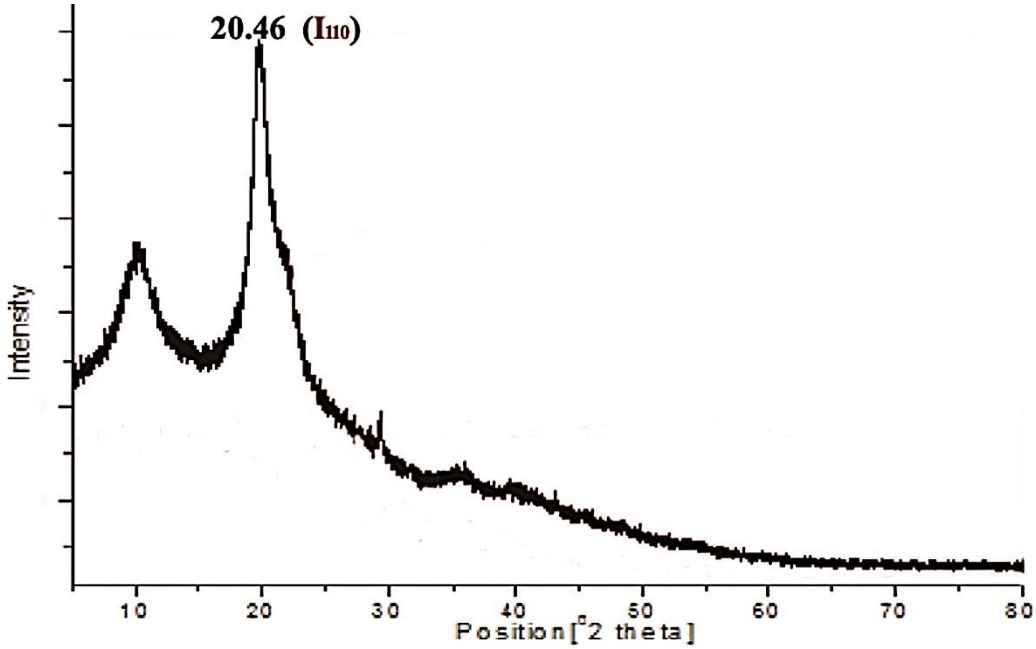

Fig 1S: X-Ray diffraction pattern of extracted chitosan

The calculation of Ct Crystallinity Index (CrI) involved the following equation [41]:

$CrI = (I_{110} - I_{am})/ I_{110}$

where I_{110} express the highest intensity (arbitrary unit) of the at 2θ diffraction peak = 19.58° and I_{am} express the 2θ amorphous diffraction intensity at 12.27°.

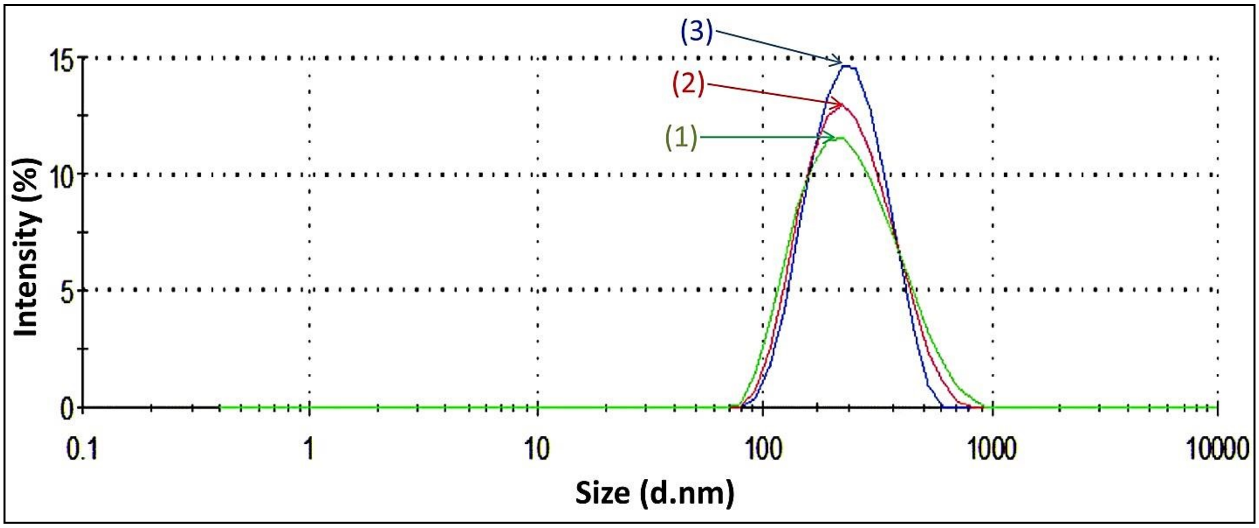

Fig 2S: Particles' size patterns (measured with DLS technique) of loaded chitosan nanoparticles with eugenol (1), curcumin (2), and their composites (3)

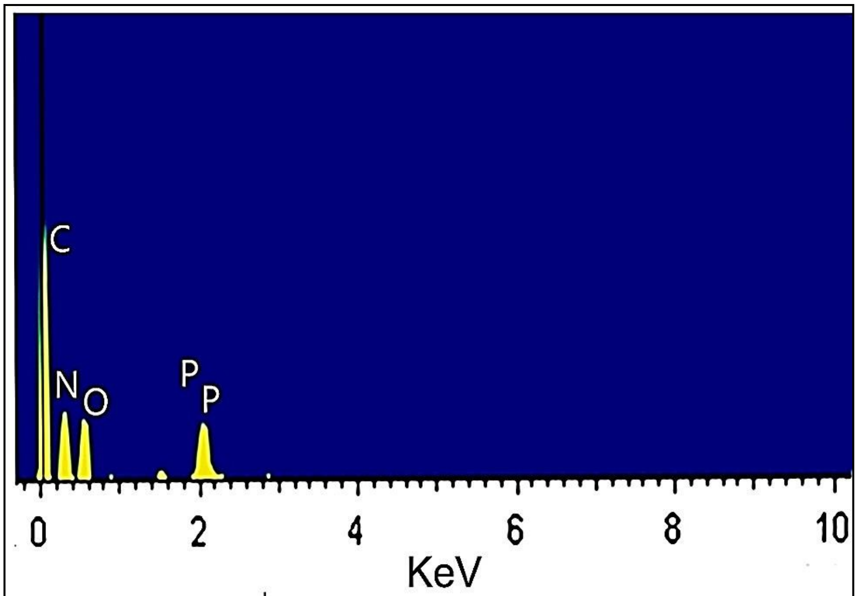

Element	Weight %	Atomic %
C K	39.98	45.36
N K	7.52	8.64
O K	42.64	40.35
P K	9.86	5.65
Total	100	

Fig 3S: EDX pattern and elemental composition of synthesized chitosan nanoparticles

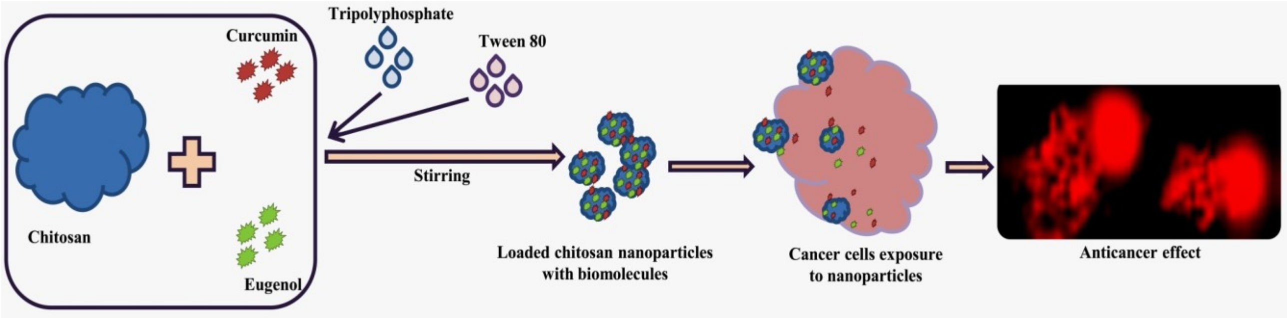

Fig 4S: Graphical abstract illustrating the synthesis method and potential anticancer mechanism of green synthesized nano composites
